# Editorial

**Published:** 1862-12

**Authors:** 


### Editorial.

### TRIAL OF WM. HOPPS FOR MURDER.

In the last number of the *Examiner* we copied so much of a report of the testimony of Dr. McFarland as exhibited the grounds on which the prisoner's council based his defence, from one of the daily newspapers of the city. If we erred in doing so, the error is more than compensated by its having elicited the following very interesting and valuable letter from Dr. McFarland, which we are sure will be read with much pleasure.

Illinois State Hospital for the Insane,

Jacksonville, *January* 17*th*, 1863.

Dr. N. S. Davis,

Dear Sir:—The last number of the *Medical Examiner*, containing what purports to be an abstract of my testimony in the Hopps Trial, reached me yesterday. So long as it stood printed in the city papers, in the form in which you have it, it gave me little concern; but when spread before the readers of your Journal, as being even any part of a rendering of an intelligible scientific testimony, I must protest, fragmentary, partial, disjointed, and patched,—I hardly recognize my own sentiments among so much of essence in the omission, and of nonsense in the supply. A very good stenographic report of all the medical testimony exists, the production of which might be of interest to the medical public, in view of the high importance of this department of medical jurisprudence. It is one of the signs of an advancing intelligence, that the plea of insanity, in criminal cases, will be set up, even in an increasing number of instances. It signifies little that prejudice and ridicule assail it, so long as the fact is well established, that it never yet has been sustained by any plural testimony of those deserving the name of experts, where subsequent facts, and a settled public opinion, did not justify the verdict given.

The position of medical science in this part of criminal jurisprudence is being completely vindicated in the history and success of Dr. RAY'S "Medical Jurisprudence of Insanity." Here is a book written by a physician, who paid no regard whatever to the *dicta* of courts in the matter to be treated of. The immutable principles of nature and disease alone governed him, in laying down what should be the path of justice, and he left courts to follow along as best they might. Follow they must, just as surely as intelligence advances among men; yet it is not a little amusing to witness the pulling back and bit-snatching of those who have got to move, whether gracefully or not. One of the incidents of every trial where this defence is set up, is the inevitable battle always to be fought over this modest man's book. Yet, however combatants may suffer, the book always comes out uppermost, with a higher vantage ground for the next contest. The twenty-five years since it appeared have witnessed an immense advance in the enlightened treatment of the plea of insanity, with, however, much yet to be accomplished.

The Hopps Trial is, in a scientific point of view, one of the highest importance. Hardly ever was the main fact clearer, yet never was a defence surrounded with greater difficulties. The fact that some of these difficulties would be removed if the State saw its duty somewhat clearer, is one reason why I wish the privilege of a slight review of the case.

William Hopps is of a family in which the proofs of hereditary insanity are so clear as not to admit of doubt. One brother, after some years of mild mania, is now demented. An adult daughter is to a degree imbecile, having some convulsive affection of the nature of chorea. Testimony which would be conclusive in a medical diagnosis, though technically ruled out of court, show his aunt insane, and throw suspicion on the sanity of prisoner's mother. Twelve years since, Hopps had intermittent, from exposure to protracted wet, in reconstructing a bridge. It was during and subsequent to that, his disease appeared. The delusion was single, and at first slight. Usually, some slight circumstance, occurring just at the time of the inception of the disease, gives a direction to the delusion which characterizes the case. In this instance, the cue was supplied by some imagined indifference of his wife to his interests, in a certain trifling arbitration in which his pride, more than his purse, suffered. This fatal direction thus given to a delusion, which otherwise might have taken any other form, was the cause of the whole chapter of woes which terminated in homicide. From this period commenced a strain of accusations of infidelity, against a woman so totally above suspicion, as not to have been entertained by any sane mind, which were continued with increasing inveteracy, till the fatal tragedy was the result. At first, her accomplices in guilt were the arbiters in the case, which went adversely, and his accusations were somewhat secret; but, as his mind became more thoroughly under the influence of the delusion, his accusations and abuse were without concealment, and the language of his reproaches more obscene and profane. Two things in relation to these accusations strike the mind as peculiarly significant. The first is: their manifestly paroxysmal character. Sometimes he appears in the evidence as showing great affection for protracted periods. This is especially the case when her fear of him has driven her from home. Then he is, at once, penitent, self-reproachful, and full of entreaties for her return. This is in accordance with the almost universal laws of mental disease. Where a delusion appends to a person or a locality, it is suspended when removed from the locality, or when the person is out of sight. This accounts for a majority of the cures wrought in an insane asylum. Where a delusion appends to a person himself, such, for instance, as the assumption of divine power, being placed in an asylum makes no difference in the progress of the disease; but, if it is a belief that his family are to starve, the chance of its cure is good as soon as they are out of his sight.

Another point in regard to these accusations, is their confinement to one person, and their always being expressed in one set of terms. As a father and neighbor he was as good as most other men. But for ten years, the sight of his wife seems to have suggested to his mind the idea of unchastity, and, eventually, the foulest pollution. He always assails her in terms implying unchastity, and never of any other vice, or even failing. Under the theory of the prosecution, that this was drunkenness simply, there is something in the fact contrary to all other experience. Who ever knew any other drunkard so special in the person, and so uniform in the terms of his vituperations? The persecuted woman herself seems to have had some idea of the cause of this, from her frequently advising him to take medicine. It does not surprise those who understand how much of a departure from a natural state has to exist in an individual before the majority of persons will regard it as insanity, that even this was viewed as a natural consequence of excessive stimulation, among his children and neighbors generally.

One of the greatest difficulties surrounding the prisoner lies in the seeming impossibility of obtaining credence for the fact that a mental disease and a vicious habit may co-exist; and to create a doubt that where the habit of intemperance and insanity exist together, they are not in relation of cause and effect, would I suspect, be an absolute impossibility? Yet the positive fact is that they *did* co-exist in this case, and were *not* in the relation of cause and effect. The prisoner had an old habit of drinking, indulged freely long before any signs of insanity existed. He drank for many years, and yet was comparatively exemplary as a husband. The abuse of his wife commences at just the date at which other circumstances fix the date of the coming on of his disease. Was there anything in the fact of the prisoner's being insane that would be likely to break up habits of intemperance? Cannot they co-exist? Does insanity mend a man's morals or reform his habits? Is a profane man less profane, a libidinous man less lecherous, or an intemperate man less intemperate after becoming insane than he was before? All experience shows that such vices are usually aggrevated when the restraints which reason supplies are taken away. So with the old habit of drinking confirmed, the effect was, that the vice and disease acted and reacted on each other. When the paroxysm was coming on, he ran to the bottle by that instinct of the disease which feeds a natural excitement with an artificial one. Had there been no such habit created, he would have been insane all the same, and with the same delusion. How much habits of intoxication modified the disease, I am not prepared to say, yet very much less than any appear to suppose. Since the old Rush theory of insanity was exploded, stimulants are perceived to have less effect on insanity than any could believe who associate it with the idea of inflammation. So naturally does paroxysmal insanity ally itself with indulgence in intoxicating drinks, that some nosologists, under the title of *Dypsomania*, recognize their co-existence as a distinct disease.

The importance of the case lies just here. Shall the incident of a habit of drinking in an insane man, be sufficient alone, and *of itself*, to take away from him that merciful leniency with which the law regards his violent acts? Is it fair to say: the man drinks, and, *therefore*, his insanity must pass for nothing? Is it not possible, in an issue of life or death, to see both states existing in the same individual, each with its distinct responsibilities? Bear in mind that the insanity of Hopps was not a consequence of drinking. Tenterden steeple, in the old story, bore the same relation to the Goodwin sands. Any skilful analyst of symptoms, and their relation to constitutions and causes, will confirm the position before laid down, that the vice and the disease were only co-existing incidents.

It might seem at first sight, that the defence is weakened for want of statements to others of the impressions he was so long conceiving. They occasionally slip from him, as in the conversation with Mr. Bradbury, Sen., fully enough to show that the declarations made just after the fatal act, and the narrations to medical experts in the jail, are consistent.

A thorough misapprehension seems to exist as to the habits of the insane in regard to their delusions. An idea prevails that the insane may readily be discovered and judged of by the expression of perverted ideas. This is true in but comparatively few instances. To one delusion among the insane that is patent and revealed, there are ten latent and concealed. The insane express their delusions far less frequently than other men disclose their ordinary motives. They are usually aware that their views will be distrusted, and they become very chary of disclosing them. But after the fatal act was committed in this instance, the delusion exhibits itself in all its force. He calls his neighbors together, asks them to witness his present sobriety, and tells them that the act has been meditated for ten years. His conduct and language during the night, while his wife lay dying in the next room, and all was horror and consternation about him, was that of the most complete self-justification. What merely drunken man, unless totally stupefied, is there who would not have been instantly sobered by such a horrible reality, and thrown into transports of remorse at what he had done? His indifference—or worse—on that night was not due to innate hardness of heart, for even the testimony not favorable to the defence, pronounces him as kind by nature as other men. What but a conviction, firmly fixed and long entertained, that the bloody deed just perpetrated was justifiable and necessary, could have endowed a man with such calmness under such circumstances? And where could have such a conviction had its birth but in the dark and mysterious chambers of mental disease?

None to whom the prisoner has given enough of his confidence since confined in the jail, to narrate the story of the last ten years of his life, for a moment doubt his insanity. Broken, scattery, and divergent, as his story is, he gives a history in which the progress of a disease is singularly plain. As is general among the insane, objects appear dark or light to him as the mental eye is obcure or clear. We see, in the story being told, those places where objects and circumstances are seen in their true, and where in their perverted, relation, according to the state of the sensorium at particular periods. While the object of his insane jealousy was pursuing an even tenor of irreproachable life, of which each day in the year was an exact counterpart of every other; he reviews her life as alternately pure by good resolutions and virtuous conduct, and black with an indiscriminate prostitution.

Could such extreme and varying views of the conduct of one whose daily life was always the same, have arisen from anything but a variation in the condition of the spectrum on which they were impressed?

I have stated that there are deficiencies in our system of juridical administration which add to the difficulties of the case. We neither have any adequate method of bringing the tests of science to bear on the investigation of the case and its elucidation before the tribunal of justice, nor have we any alternative in such a case as Hopps', between absolute acquittal and discharge, and the penalty of death. In the defence on a plea of insanity, the expert witnesses stand in the bad light of partisans. They are not allowed the privilege of neutrality. In most of our courts they are sworn in the same motley group with witnesses of fact, and it is a chance if they do not have to stand the same pettifogging fire on a cross-examination, to which those luckless unfortunates are yet subject, notwithstanding the march of refinement everywhere else.

Sterne was not the last who must say: "They order these matters better in France." There, when the line of defence is adopted in a case of alleged insanity, the prisoner is removed to where he can be subjected to skilful *espionage* by those who have something at stake in rendering an opinion. In that way the tests of a mental condition become infallible. Now, what to our shame, as the respecters and conservators of justice, have we seen in Hopps' case? Intoxicating liquor, apparently without stint or limit, is supplied the prisoner *by the demijohn*, when all being dark both behind and before him,—every manly incentive withdrawn,—he has no earthly inducement to keep above the brute! And, as if to show that a lower depth of cruelty and baseness could be reached, *the liquor account-book is brought into court, and read by the doggery-keeper who owned it*, item by item,—so many X's to the ale, and so much proof to the whiskey,—to show, on the part of the prosecution, how much of specific liquors was necessary to drown the horrors of the man whenever a gleam of reason enabled him to see whatever was black behind and cavernous before him! What a preparation for a trial where life or death is the issue! Yet it is through such scenes as these, that reforms in the administration of an enlightened justice, have to come.

Another very great evil in doing exact justice to such a case, is, that neither in our laws nor in the provisions made by the State for the exigencies of an enlightened judicial administration, is there any halting-place, where an atrocious homicide has been committed, even in cases where insanity is well proven, between the scaffold and an absolute acquittal, with full liberty afterwards. The most strenuous advocate of a justice made to conform to the conditions of mental disease, holds that where a life has been sacrificed, the offender should be permanently placed beyond the possibility of further inroads on the safety of persons or property. The manner of this isolation, as we are at present circumstanced, is attended with great difficulties. Those who have in charge the interests of institutions for the insane, protest in the most emphatic terms, against their admission among those whose misfortune should not be enhanced by the compelled presence, of persons having the taint of a bloody crime upon them. Every consideration of humanity supports this protest. The shedding of blood seems to beget, in one not supported by the aid of reason, that tiger-like instinct which craves the effusion of still more; and persons who have committed homicide, and been acquitted on the ground of insanity, are objects of especial dread by those having the general custody of the insane, from the frequent activity of this dreadful instinct.

Ordinary institutions for the insane have not the elements of custodial security about them demanded for such cases. All their appliances for the restraining of liberty are made as mild and little offensive as possible. Escapes from them are frequent; —a less evil than a multiplication of prison-like restraints. All these considerations, without doubt, suggest themselves to a conscientious juryman, where an aggravated homicide is defended on the ground of insanity. He justly dreads the possession of liberty by one proven to have taken a life without compunction, and yet, by the terms of his oath, must render a verdict of unqualified acquittal, or must visit upon the prisoner at the bar the extreme penalties of the law. He will, almost as a matter of course, render a verdict without regard to the plea made, and leave to the executive the care of making investigation and modifying the sentence as the facts may demand. He expects the prisoner, if insane, to reach the lunatic asylum by the somewhat circuitous route of a gallows sentence, and a penitentiary probation. Now, this is all wrong, and indicates a deficiency in the machinery of a thorough administration of justice. If the plea of insanity is at all recognized, some means should be devised to place the accused, before trial, where he can be the subject of an intelligent scrutiny, for a sufficient time to establish the fact. Then, if the defence be sustained, the place of scrutiny should become the place of sentence, where, in common with all persons found insane and transferred from penitentiaries, he should be kept for life, or a lesser period, as the case may be. This system is already initiated in the leading commonwealths of the country. The report of the New York Asylum for Insane Convicts, at Auburn, for 1860, shows more than fifty inmates,—and those consisting entirely of persons transferred from the different State penitentiaries. The number will be largely increased when the contemplated system is so far completed that the institution will have a direct connection with the courts, as it still appears not to have.

The want of some such system as this, makes the duty of experts in cases defended on this plea, an especially unwelcome, and sometimes unnecessary, one. It seems in some cases almost useless to put in this highest of all defenses, when the essentials of safety to the public are entirely wanting, except as they are reached by the indirect manner which we have indicated, and which has to run quite around the plea which science sets up. These considerations are presented in connection with this case, to the attention of the medical profession, to whom all such interests are entrusted.

Very respectfully Yours,

AND. McFARLAND.

### DEATH OF DR. GEORGE COATSWORTH.

We have received the following communication from the officers of the 88th Regt. of Ill. Vols., in relation to their late surgeon. Dr. COATSWORTH was a resident well known in this city:—

The officers of the 88th Regt., Ill. Vols., deeply feeling the loss of their surgeon, Dr. GEORGE COATSWORTH, who died of pneumonia at Murfreesbro, Tenn., January 9th, 1863, were called together by Col. Sherman, when a committee, consisting of Lieut.-Col. Chadbourne, Capt. Sheridan, Capt. McClery, and Lieut. Bigelow, was selected to draft resolutions expressive of their feelings.

Col. Chadbourne, on presenting the resolutions, remarked that he did not feel that the occasion was an ordinary one, that he was opposed to the usual way of calling meetings and passing the customary resolutions. He believed that every one present felt the loss of a true friend in the death of Dr. COATSWORTH.

The resolutions as passed were as follows:—

Whereas Providence has seen fit to remove from us our surgeon GEORGE COATSWORTH by death.

Resolved, that we the officers of this regiment tender to his family our heart-felt sympathy in this their sudden bereavement. We bear willing tribute to his many excellencies of character, and his greatness of head and heart. To us his death is an irreparable loss, and to the profession of which he was so able a member. In our friend we recognize a man of more than ordinary ability and attainments. Our respect and love for him increased, as a continual daily association with him developed those traits of character which a less intimate association would fail to discern. We feel that not only has the regiment by his death lost a true friend and skilful surgeon, but the profession one of its clearest thinkers, most devoted students, and accomplished operators. But though the loss is hard to bear, we find relief in the fact that he died in the noblest way a man can die:—at his post in the laborious performance of his duty.

F. F. SHERMAN, *Chairman.*

J. SEYMOUR BALLARD, *Sec'y.*

Explanations:—In this and the preceding number we have occupied more space with selected matter than usual, and have filled nearly all of it with the very interesting and valuable monograph of Prof. A. Flint, on *Cholesterine.* The reference to plates made in the article, refer to plates accompanying the article as published in the *American Journal of Medical Sciences, but which of course we could not copy.*

The Future.—Owing to the absence, in the service of the army, of many of our assistants and contributors, we have not been able to keep up the usual supply of Clinical reports, or to publish the *Examiner* promptly on time, during the last six months. This difficulty is now remedied, and we shall soon issue the *Examiner* again on time, and with its *Clinical* department as well filled as is desirable.

Prof. E. Andrews; this able surgeon, who has been constantly on active duty as surgeon to the 1st Regt. Ill. Artillery for the last nine months, has returned and resumed his professional duties, both in the University and in private practice.

Change in a well-known Drug Store.—We would invite the attention of our readers to the advertisement of Messrs. Bliss & Sharp, who have recently purchased from J. H. Reed & Co. their well-known Retail Drug Store at 144 Lake Street. They have made such alterations as will enable them to pay particular attention to physicians' orders. Mr. Bliss has been a member of the firm of J. H. Reed & Co. for the past eight years, and consequently, needs no commendation to the patrons of the late firm.

---

(CIRCULAR No. 13.)

Surgeon-General's Office,
Washington, *December* 5, 1862.

1. The attention of medical directors is called to the numerous cases of neglect in the transmission of the Weekly Report of Hospitals, and the Monthly Report of sick and wounded.

In future they will require medical officers in charge of hospitals to forward to their office the Weekly Hospital Report, on the last day of each week; and they will promptly forward them to this office, accompanied by a list of such officers as have neglected this duty.

They will also require the Monthly Report of sick and wounded to be forwarded to them, and will transmit them to this office, duly filled up, as to date and place, and also accompanied by a list of names of those officers who may have failed to forward these required reports.

Medical directors will see that the surgeons under their direction are kept duly supplied with blank forms necessary for the above reports.

2. Medical directors having supervision of several General Hospitals, will require from the surgeon in charge of each hospital, daily report of such changes as may have taken place during the preceding day. This report will state the name, company, and regiment of each soldier admitted, returned to duty, discharged, transferred to other hospitals, died, etc. etc., and these names will, from time to time, be recorded in a book kept for that purpose in the office of the medical director.

Every facility will be afforded the agents of the Sanitary Commission, and the friends of sick and wounded soldiers, in procuring such information concerning the inmates of hospitals, as they may, from time to time, desire.

3. Surgeons in charge of General Hospitals will, upon receipt of this circular, report to the commanders of companies the names of any soldiers of their company deceased, or discharged from the service, while in their hospital, and concerning whom these reports have not been duly made.

These reports will strictly conform to paragraphs 152 and 170, General Regulations, to which, for the future, particular attention must be paid.

4. Persons detailed for duty in any capacity in General Hospitals, by medical officers in charge, without proper authority, will not be recognized at this office as hospital employés, and medical officers so employing them will be personally responsible for the wages due them.

They will also be held pecuniarily responsible for any payment over their signature made to cooks and laundresses in excess of the number authorized by regulations to the hospital under their charge.

5. Medical officers are explicitly informed that regulations on the above subjects have been written and published to be observed by them, and the various infractions which, from time to time, they have allowed themselves to make, have not been overlooked in the past, nor will be for the future.

W. A. Hammond, Surgeon-General.

